# *catena*-Poly[[(5,5′-dimethyl-2,2′-bi­pyridine-κ^2^ *N*,*N*′)cadmium(II)]-di-μ-iodido]

**DOI:** 10.1107/S1600536810014091

**Published:** 2010-04-24

**Authors:** Roya Ahmadi, Khadijeh Kalateh, Vahid Amani

**Affiliations:** a Islamic Azad University, Shahr-e-Rey Branch, Tehran, Iran

## Abstract

In the title coordination polymer, [CdI_2_(C_12_H_12_N_2_)]_*n*_, the Cd^2+^ ion lies on a twofold rotation axis: it is six-coordinated in a distorted *cis*-CdN_2_I_4_ octa­hedral geometry by two N atoms from a chelating 5,5′-dimethyl-2,2′-bipyridine ligands and four bridging iodide anions. The bridging function of the iodide ions leads to a chain structure propagating in [001].

## Related literature

For related structures, see: Ahmadi *et al.* (2008 ▶); Albada *et al.* (2004 ▶); Amani *et al.* (2007 ▶, 2009 ▶); Chattopadhyay *et al.* (2008 ▶); Guo *et al.* (2006 ▶); Kalateh *et al.* (2008 ▶, 2010 ▶); Khalighi *et al.* (2008 ▶); Maheshwari *et al.* (2007 ▶); Tadayon Pour *et al.* (2008 ▶); Yu *et al.* (2007 ▶).
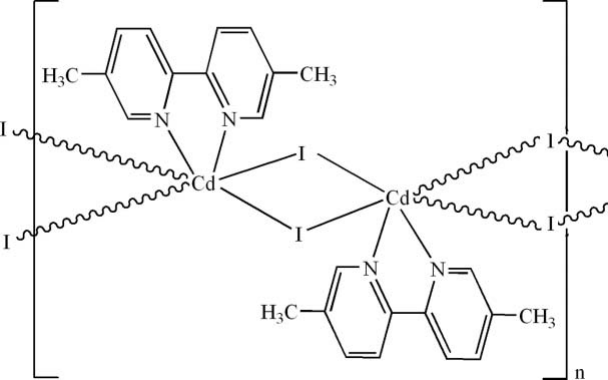


## Experimental

### 

#### Crystal data

[CdI_2_(C_12_H_12_N_2_)]
*M* *_r_* = 550.45
Monoclinic, 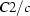

*a* = 19.086 (4) Å
*b* = 10.057 (2) Å
*c* = 7.8451 (16) Å
β = 101.80 (3)°
*V* = 1474.0 (5) Å^3^
*Z* = 4
Mo *K*α radiation
μ = 5.65 mm^−1^
*T* = 298 K
0.25 × 0.15 × 0.12 mm

#### Data collection

Bruker SMART CCD diffractometer
Absorption correction: multi-scan (*SADABS*; Bruker, 1998 ▶) *T*_min_ = 0.380, *T*_max_ = 0.510
8294 measured reflections
1981 independent reflections
1832 reflections with *I* > 2σ(*I*)
*R*_int_ = 0.062

#### Refinement

*R*[*F* ^2^ > 2σ(*F* ^2^)] = 0.037
*wR*(*F* ^2^) = 0.098
*S* = 1.23
1981 reflections
79 parameters
H-atom parameters constrained
Δρ_max_ = 1.30 e Å^−3^
Δρ_min_ = −1.43 e Å^−3^

### 

Data collection: *SMART* (Bruker, 1998 ▶); cell refinement: *SAINT* (Bruker, 1998 ▶); data reduction: *SAINT*; program(s) used to solve structure: *SHELXTL* (Sheldrick, 2008 ▶); program(s) used to refine structure: *SHELXTL*; molecular graphics: *SHELXTL*; software used to prepare material for publication: *WinGX* (Farrugia, 1999 ▶).

## Supplementary Material

Crystal structure: contains datablocks I, global. DOI: 10.1107/S1600536810014091/hb5403sup1.cif

Structure factors: contains datablocks I. DOI: 10.1107/S1600536810014091/hb5403Isup2.hkl

Additional supplementary materials:  crystallographic information; 3D view; checkCIF report

## Figures and Tables

**Table 1:** Selected bond lengths (Å)

Cd1—N1	2.347 (3)
Cd1—I1	2.8586 (7)
Cd1—I1^i^	3.1628 (8)

Symmetry code: (i) 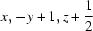.

**Fig. 1.**
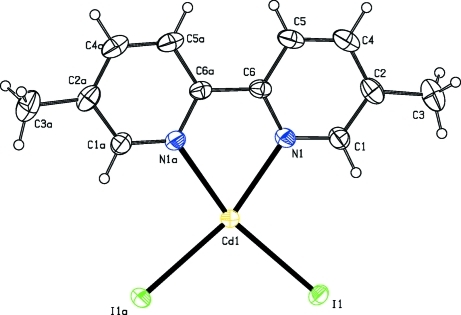
Fragment of a polymeric chain in (I) with displacement ellipsoids drawn at the 50% probability level. [Symmetry code: (a) -x+1, y, -z+5/2].

## supplementary crystallographic information

### Comment

In a recent paper, we reported the synthes and crystal structure of [Cd(5,5'-dmbpy)(µ-Cl)_2_]_n_, (Ahmadi *et al.*, 2008) and [Cd(4,4'-dmbpy)(DMSO)I_2_], (Kalateh *et al.*, 2010) [where 5,5'-dmbpy is 5,5'-dimethyl-2,2'-bipyridine and 4,4'-dmbpy is 4,4'-dimethyl-2,2'-bipyridine].

5,5'-Dimethyl-2,2'-bipyridine (5,5'-dmbipy), is a good bidentate ligand, and numerous complexes with 5,5'-dmbipy have been prepared, such as that of zinc (Khalighi *et al.*, 2008), indium (Kalateh *et al.*, 2008), iron (Amani *et al.*, 2007), platin (Amani *et al.*, 2009; Maheshwari *et al.*, 2007), copper (Albada *et al.*, 2004) and mercury (Tadayon Pour *et al.*, 2008).

There are several Cd^II^ polymer complexes, with formula, [Cd(N—N)(µ-I)_2_]_n_, such as [Cd(phen)(µ-I)_2_]_n_, (Guo *et al.*, 2006), [Cd(bipy)(µ-I)_2_]_n_, (Yu *et al.*, 2007) and [Cd(ampy)(µ-I)_2_]_n_, (Chattopadhyay *et al.*, 2008) [where phen is 1,10-phenanthroline , bipy is 2,2'-bipyridine and ampy is 2-aminomethylpyridine] have been synthesized and characterized by single-crystal X-ray diffraction methods. Here, we report the synthesis and structure of the title compound.

The asymmetric unit of the title compound, (Fig. 1), contains one half -molecule; a twofold rotation axis passes through the Cd atom. The Cd^II^ atom is six-coordinated in a distorted octahedral configuration by two N atoms from 5,5'-dimethyl-2,2'-bipyridine and four bridging I atoms. The bridging function of the iodo atoms leads to a one-dimensional chain structure. The Cd—I and Cd—N bond lengths and angles (Table 1) are within normal range [Cd(phen)(µ-I)_2_]_n_, (Guo *et al.*, 2006) and [Cd(bipy)(µ-I)_2_]_n_, (Yu *et al.*, 2007).

### Experimental

A solution of 5,5'-dimethyl-2,2'-bipyridine (0.25 g, 1.33 mmol) in methanol (10 ml) was added to a solution of CdI_2_ (0.49 g, 1.33 mmol) in methanol (10 ml) at room temperature. Colourless blocks of (I) were obtained by methanol diffusion to a colorless solution in DMSO. Suitable crystals were isolated after one week (yield; 0.52 g, 71.0%).

### Refinement

All H atoms were positioned geometrically, with C—H = 0.93Å and constrained to ride on their parent atoms, with U_iso_(H) = 1.2U_eq_.

### Crystal data

**Table:**

[CdI_2_(C_12_H_12_N_2_)]	*F*(000) = 1008
*M**_r_* = 550.45	*D*_x_ = 2.480 Mg m^−^^3^
Monoclinic, *C*2/*c*	Mo *K*α radiation, λ = 0.71073 Å
Hall symbol: -C 2yc	Cell parameters from 351 reflections
*a* = 19.086 (4) Å	θ = 2.2–29.3°
*b* = 10.057 (2) Å	µ = 5.65 mm^−^^1^
*c* = 7.8451 (16) Å	*T* = 298 K
β = 101.80 (3)°	Block, colorless
*V* = 1474.0 (5) Å^3^	0.25 × 0.15 × 0.12 mm
*Z* = 4	

### Data collection

**Table:**

Bruker SMART CCD diffractometer	1981 independent reflections
Radiation source: fine-focus sealed tube	1832 reflections with *I* > 2σ(*I*)
graphite	*R*_int_ = 0.062
phi and ω scans	θ_max_ = 29.3°, θ_min_ = 2.2°
Absorption correction: multi-scan (*SADABS*; Bruker, 1998)	*h* = −26→26
*T*_min_ = 0.380, *T*_max_ = 0.510	*k* = −13→12
8294 measured reflections	*l* = −10→10

### Refinement

**Table:**

Refinement on *F*^2^	Primary atom site location: structure-invariant direct methods
Least-squares matrix: full	Secondary atom site location: difference Fourier map
*R*[*F*^2^ > 2σ(*F*^2^)] = 0.037	Hydrogen site location: inferred from neighbouring sites
*wR*(*F*^2^) = 0.098	H-atom parameters constrained
*S* = 1.23	*w* = 1/[σ^2^(*F*_o_^2^) + (0.0571*P*)^2^ + 0.4175*P*] where *P* = (*F*_o_^2^ + 2*F*_c_^2^)/3
1981 reflections	(Δ/σ)_max_ = 0.037
79 parameters	Δρ_max_ = 1.30 e Å^−^^3^
0 restraints	Δρ_min_ = −1.43 e Å^−^^3^

### Special details

**Table:**

Geometry. All esds (except the esd in the dihedral angle between two l.s. planes) are estimated using the full covariance matrix. The cell esds are taken into account individually in the estimation of esds in distances, angles and torsion angles; correlations between esds in cell parameters are only used when they are defined by crystal symmetry. An approximate (isotropic) treatment of cell esds is used for estimating esds involving l.s. planes.
Refinement. Refinement of *F*^2^ against ALL reflections. The weighted *R*-factor wR and goodness of fit *S* are based on *F*^2^, conventional *R*-factors *R* are based on *F*, with *F* set to zero for negative *F*^2^. The threshold expression of *F*^2^ > σ(*F*^2^) is used only for calculating *R*-factors(gt) etc. and is not relevant to the choice of reflections for refinement. *R*-factors based on *F*^2^ are statistically about twice as large as those based on *F*, and *R*- factors based on ALL data will be even larger.

### Fractional atomic coordinates and isotropic or equivalent isotropic displacement parameters (Å^2^)

**Table:**

	*x*	*y*	*z*	*U*_iso_*/*U*_eq_	
C1	0.3791 (2)	0.7656 (5)	1.0047 (5)	0.0482 (9)	
H1	0.3615	0.6825	0.9652	0.058*	
C2	0.3413 (3)	0.8765 (5)	0.9329 (6)	0.0546 (11)	
C3	0.2732 (3)	0.8623 (8)	0.7991 (8)	0.0758 (17)	
H3A	0.2792	0.9020	0.6917	0.114*	
H3B	0.2350	0.9062	0.8395	0.114*	
H3C	0.2619	0.7698	0.7807	0.114*	
C4	0.3706 (3)	0.9988 (5)	0.9920 (6)	0.0590 (12)	
H4	0.3478	1.0767	0.9473	0.071*	
C5	0.4327 (3)	1.0059 (5)	1.1151 (6)	0.0545 (10)	
H5	0.4521	1.0881	1.1531	0.065*	
C6	0.4666 (2)	0.8885 (4)	1.1833 (5)	0.0398 (8)	
N1	0.43894 (18)	0.7698 (3)	1.1264 (4)	0.0414 (7)	
Cd1	0.5000	0.57933 (4)	1.2500	0.04719 (14)	
I1	0.407345 (14)	0.39302 (3)	1.03947 (3)	0.04393 (12)	

### Atomic displacement parameters (Å^2^)

**Table:**

	*U*^11^	*U*^22^	*U*^33^	*U*^12^	*U*^13^	*U*^23^
C1	0.049 (2)	0.048 (2)	0.0463 (19)	0.0039 (18)	0.0055 (16)	0.0064 (16)
C2	0.050 (2)	0.066 (3)	0.051 (2)	0.013 (2)	0.0171 (18)	0.019 (2)
C3	0.054 (3)	0.100 (5)	0.071 (3)	0.012 (3)	0.005 (2)	0.027 (3)
C4	0.074 (3)	0.049 (3)	0.058 (2)	0.021 (2)	0.023 (2)	0.015 (2)
C5	0.077 (3)	0.035 (2)	0.055 (2)	0.012 (2)	0.023 (2)	0.0084 (17)
C6	0.051 (2)	0.0328 (18)	0.0388 (17)	0.0020 (14)	0.0168 (15)	0.0021 (12)
N1	0.0470 (17)	0.0366 (17)	0.0400 (14)	0.0018 (13)	0.0074 (12)	0.0052 (12)
Cd1	0.0564 (3)	0.0300 (2)	0.0463 (2)	0.000	−0.01021 (18)	0.000
I1	0.04992 (19)	0.03831 (18)	0.04068 (17)	−0.00834 (9)	0.00254 (11)	−0.00433 (8)

### Geometric parameters (Å, °)

**Table:**

C1—N1	1.331 (6)	C5—H5	0.9300
C1—C2	1.384 (6)	C6—N1	1.344 (5)
C1—H1	0.9300	C6—C6^i^	1.474 (9)
C2—C4	1.391 (8)	Cd1—N1	2.347 (3)
C2—C3	1.501 (8)	Cd1—N1^i^	2.347 (3)
C3—H3A	0.9600	Cd1—I1	2.8586 (7)
C3—H3B	0.9600	Cd1—I1^i^	2.8586 (7)
C3—H3C	0.9600	Cd1—I1^ii^	3.1628 (8)
C4—C5	1.369 (9)	Cd1—I1^iii^	3.1629 (8)
C4—H4	0.9300	I1—Cd1^iii^	3.1629 (8)
C5—C6	1.399 (6)		
			
N1—C1—C2	124.4 (5)	C5—C6—C6^i^	122.5 (3)
N1—C1—H1	117.8	C1—N1—C6	119.2 (4)
C2—C1—H1	117.8	C1—N1—Cd1	123.4 (3)
C1—C2—C4	115.8 (5)	C6—N1—Cd1	117.3 (3)
C1—C2—C3	120.8 (5)	N1^i^—Cd1—N1	70.55 (18)
C4—C2—C3	123.3 (5)	N1^i^—Cd1—I1	165.97 (9)
C2—C3—H3A	109.5	N1—Cd1—I1	95.74 (9)
C2—C3—H3B	109.5	N1^i^—Cd1—I1^i^	95.74 (9)
H3A—C3—H3B	109.5	N1—Cd1—I1^i^	165.97 (9)
C2—C3—H3C	109.5	I1—Cd1—I1^i^	98.09 (3)
H3A—C3—H3C	109.5	N1^i^—Cd1—I1^ii^	86.26 (8)
H3B—C3—H3C	109.5	N1—Cd1—I1^ii^	85.51 (8)
C5—C4—C2	120.9 (4)	I1—Cd1—I1^ii^	95.844 (16)
C5—C4—H4	119.6	I1^i^—Cd1—I1^ii^	90.771 (16)
C2—C4—H4	119.6	N1^i^—Cd1—I1^iii^	85.51 (8)
C4—C5—C6	119.4 (5)	N1—Cd1—I1^iii^	86.26 (8)
C4—C5—H5	120.3	I1—Cd1—I1^iii^	90.771 (16)
C6—C5—H5	120.3	I1^i^—Cd1—I1^iii^	95.842 (16)
N1—C6—C5	120.2 (4)	I1^ii^—Cd1—I1^iii^	169.91 (2)
N1—C6—C6^i^	117.4 (2)	Cd1—I1—Cd1^iii^	89.229 (16)
			
N1—C1—C2—C4	1.8 (7)	C6—N1—Cd1—N1^i^	−0.26 (19)
N1—C1—C2—C3	−178.5 (4)	C1—N1—Cd1—I1	2.9 (3)
C1—C2—C4—C5	−0.7 (7)	C6—N1—Cd1—I1	−177.2 (3)
C3—C2—C4—C5	179.6 (5)	C1—N1—Cd1—I1^i^	−167.5 (2)
C2—C4—C5—C6	−0.5 (7)	C6—N1—Cd1—I1^i^	12.3 (5)
C4—C5—C6—N1	0.9 (6)	C1—N1—Cd1—I1^ii^	−92.5 (3)
C4—C5—C6—C6^i^	−179.7 (4)	C6—N1—Cd1—I1^ii^	87.4 (3)
C2—C1—N1—C6	−1.5 (6)	C1—N1—Cd1—I1^iii^	93.3 (3)
C2—C1—N1—Cd1	178.4 (3)	C6—N1—Cd1—I1^iii^	−86.8 (3)
C5—C6—N1—C1	0.1 (6)	N1^i^—Cd1—I1—Cd1^iii^	74.4 (3)
C6^i^—C6—N1—C1	−179.4 (4)	N1—Cd1—I1—Cd1^iii^	86.32 (8)
C5—C6—N1—Cd1	−179.8 (3)	I1^i^—Cd1—I1—Cd1^iii^	−96.013 (15)
C6^i^—C6—N1—Cd1	0.7 (5)	I1^ii^—Cd1—I1—Cd1^iii^	172.370 (15)
C1—N1—Cd1—N1^i^	179.8 (4)	I1^iii^—Cd1—I1—Cd1^iii^	0.0

Symmetry codes: (i) −*x*+1, *y*, −*z*+5/2; (ii) *x*, −*y*+1, *z*+1/2; (iii) −*x*+1, −*y*+1, −*z*+2.
